# Trends in Mortality Rate from Cardiovascular Disease in Brazil, 1980-2012

**DOI:** 10.5935/abc.20160077

**Published:** 2016-07

**Authors:** Antonio de Padua Mansur, Desidério Favarato

**Affiliations:** Instituto do Coração (InCor) - HC FMUSP, São Paulo, SP - Brazil

**Keywords:** Cardiovascular Diseases / epidemiology, Mortality / trends, Myocardial Ischemia, Brain Ischemia.

## Abstract

**Background:**

Studies have questioned the downward trend in mortality from cardiovascular diseases (CVD) in Brazil in recent years.

**Objective:**

to analyze recent trends in mortality from ischemic heart disease (IHD) and stroke in the Brazilian population.

**Methods:**

Mortality and population data were obtained from the Brazilian Institute of Geography and Statistics and the Ministry of Health. Risk of death was adjusted by the direct method, using as reference the world population of 2000. We analyzed trends in mortality from CVD, IHD and stroke in women and men in the periods of 1980-2006 and 2007-2012.

**Results:**

there was a decrease in CVD mortality and stroke in women and men for both periods (p < 0.001). Annual mortality variations for periods 1980-2006 and 2007-2012 were, respectively: CVD (total): -1.5% and -0.8%; CVD men: -1.4% and -0.6%; CVD women: -1.7% and -1.0%; DIC (men): -1.1% and 0.1%; stroke (men): -1.7% and -1.4%; DIC (women): -1.5% and 0.4%; stroke (women): -2.0% and -1.9%. From 1980 to 2006, there was a decrease in IHD mortality in men and women (p < 0.001), but from 2007 to 2012, changes in IHD mortality were not significant in men [y = 151 + 0.04 (R^2^ = 0.02; p = 0.779)] and women [y = 88-0.54 (R^2^ = 0.24; p = 0.320).

**Conclusion:**

Trend in mortality from IHD stopped falling in Brazil from 2007 to 2012.

## Introduction

Cardiovascular diseases (CVD) are the main cause of death in the Brazilian population.^1,2^ CVDs are responsible for at least 20% of deaths in our population over 30 years old. In the South and Southeast regions of the country, the rate of mortality from CVD was even greater than in other regions.^3^ Previous studies have shown consistent data about the downward trend in the mortality rate from CVD in Brazil.^4,5^ Deaths by cerebrovascular diseases (CBVD) had greater reduction in mortality rate when compared to ischemic heart disease (IHD).^4^ A recent update in CVD mortality data in Brazil and in the metropolitan region of São Paulo showed a downward trend in the rate of mortality from IHD and CBVD between 1990 and 2009.^2^ In Brazil, starting in 1987, deaths from IHD were, in men, higher than deaths from CBVD. In women, however, this difference was only noticed as of 1999. Both causes of death showed a downward trend in the period of 1980 - 2009, but this trend was more evident in deaths from CBVD. However, in men, from 2007 to 2009, the mortality rate adjusted by age from IHD remained unaltered. Nevertheless, due to the period of only three years, it was not possible to establish a real trend in mortality. This study updated the rate of mortality from cardiovascular diseases in Brazil until 2012. Trends in rate of mortality from CVD, IHD and CBVD in the period of 2007-2012 were also analysed and compared to previous years.

## Methods

Rates of mortality from CVD, IHD and CBVD, in Brazil, from 1980 to 2012 and in the periods of 1980-2006 and 2007-2012 were analysed. The data about mortality were obtained on the Ministry of Health of Brazil's website www.datasus.gov.br. Population data, from the Brazilian institute of Geography and Statistics (IBGE), were obtained on the same website. Deaths from 1990 to 1995 were classified according to ICD-9, 9th Review Conference of the International Classification of Diseases (ICD) of 1975, adopted by the 20th World Health Assembly. Starting in 1996, the mortality data were obtained in the 10^th^ review of the International Classification of Diseases. Diseases of the circulatory system (CDs) were grouped in codes 390 to 459, IHDs in codes 410 to 414, and CBVDs in codes 430 to 438, in the 9^th^ review of the ICD. Mortality data, starting in 1996, was classified by the 10^th^ review of the ICD. CDs are grouped in codes 100-199, IHDs in codes 120 to 125, and CBVDs in codes 160 to 169. Mortality after 30 years of age, according to gender, per 100,000 inhabitants, was analysed in the following age groups: 30-39 years of age; 40-49 years of age; 50-59 years of age; 60-69 years of age; 70-79 years of age and ≥ 80 years of age. For comparison, mortality was adjusted by direct method for the age according to standard world population of the year 2000.^6^ Simple linear regression was used to analyse the temporal evolution of mortality rate associated to CVD, IHD, and CBVD, followed by the comparison of slopes of regression lines. The level of significance was p < 0.05. The statistical software used was Primer of Biostatistics, version 4.02.9.^7^

## Results

**Figure 1:**
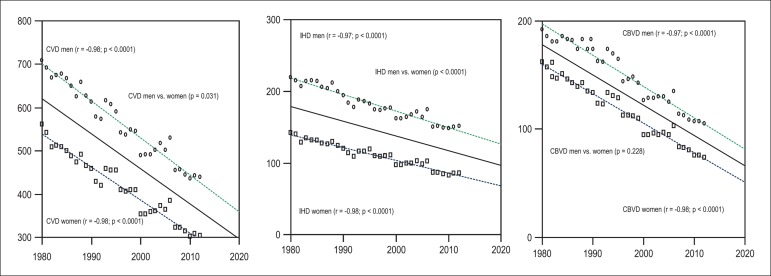
Analysis of the simple linear regression and comparison between the lines of regression of mortality from cardiovascular diseases (CVD), ischemic heart diseases (IHD), and cerebrovascular diseases (CBVD) between men and women, in the period of 1980 to 2012.

From 1980 to 2012, there was a decrease in mortality from CVD, IHD and CBVD in men and women (Table 1). The results of the linear regression for such period were: CVD total: y = 627.4-8.0 (R^2^ = 0.98. p < 0.001), CVD men: y = 684.4-8.6 (R^2^ = 0.94. p < 0.001), CVD women: y = 593.9-7.6 (R^2^ = 0.95. p < 0.001), IHD total: y = 181-2.0 (R^2^ = 0.23. p < 0.001), IHD men: y = 219.2-2.3 (R^2^ = 0.93, p < 0.001), IHD women: y = 144.9-1.8 (R^2^ = 0.95. p < 0.001), CBVD total: y = 181.3-2.8 (R^2^ = 0.69, p < 0.001), CBVD men: y = 231.6-2.9 (R^2^ = 0.91, p < 0.001), CBVD women: y = 171.3-2.7 (R^2^ = 0.94, p < 0.001). The comparison of linear regressions between men and women showed a larger reduction in mortality from CVD (p = 0.031) and IHD (p < 0.001) in men, but no significant reduction in CBVD (p = 0.228) (Figure 1). The percentage of reduction of death from CVD, IHD, and CBVD between 1980-2012, 1980-2006 and 2007-2012 are shown in Table 2.

**Table 1:** Risk of death* from cardiovascular diseases (CVD), ischemic heart disease (IHD), and cerebrovascular diseases (CBVD), per 100.000 inhabitants, and the total variation in the period of observation (1980-2012), in men (M) and women (W), in Brazil.

Year	CVD	CVD men	CVD women	IHD	CBVD	IHD men	CBVD men	IHD women	CBVD women
1980	636	709	562	181	178	220	193	143	163
1981	618	691	544	178	172	215	186	141	157
1982	589	669	510	168	165	207	181	129	149
1983	594	675	513	175	164	214	182	136	147
1984	593	677	510	174	169	215	187	132	151
1985	585	667	502	174	165	215	184	133	147
1986	569	650	488	167	163	206	183	128	144
1987	550	626	474	166	157	204	175	128	140
1988	576	660	492	172	164	213	183	131	144
1989	547	628	466	162	155	200	175	125	136
1990	536	613	459	158	155	195	174	120	135
1991	504	579	429	150	144	185	163	115	125
1992	497	573	421	144	143	179	162	110	124
1993	538	618	458	152	155	189	175	116	134
1994	532	609	455	151	151	187	170	116	132
1995	524	591	457	152	147	184	165	119	130
1996	477	543	411	143	129	176	145	110	114
1997	472	538	407	141	130	173	147	109	113
1998	481	550	411	144	131	177	149	110	112
1999	478	546	411	144	127	178	144	111	110
2000	423	491	356	130	111	162	127	97	95
2001	423	492	354	130	113	162	130	97	95
2002	426	491	360	132	113	164	130	99	97
2003	433	503	362	133	113	167	131	100	96
2004	447	519	375	138	114	172	131	103	97
2005	434	503	365	132	111	165	126	98	96
2006	458	530	386	139	119	174	136	103	103
2007	391	457	325	120	99	151	115	88	84
2008	391	458	324	120	98	153	114	88	83
2009	380	446	315	117	95	149	110	85	81
2010	370	438	302	116	92	148	107	83	76
2011	377	445	309	119	92	152	108	86	76
2012	372	440	305	119	90	152	105	86	75
%var	-41	-38	-46	-34	-49	-31	-45	-40	-54

* adjusted by direct method for the standard world population in 2000 , var(%) = percentage variation (2012/1980).

**Table 2:** Percentage variation of the risk of death adjusted for the age of cardiovascular diseases (CVD), ischemic heart diseases (IHD) and cerebrovascular diseases (CBVD) from 1980 to 2012, and for the periods of 1980 to 2006 and 2007 to 2012

Periods	CVD	CVD men	CVD women	IHD	CBVD	IHD men	CBVD men	IHD men	CBVD women
1980-2012	-59	-62	-54	-66	-51	-69	-55	-60	-46
1980-2006	-28	-25	-31	-23	-33	-21	-30	-28	-37
2007-2012	-5	-4	-6	0	-10	1	-8	-2	-11
1980-2012/year	-1	-1	-1	-1	-1	-1	-1	-1	-2
1980-2006/year	-1	-1	-1	-1	-1	-1	-1	-1	-1
2007-2012/year	-1	-1	-1	0	-2	0	-1	0	-2

**Figure 2:**
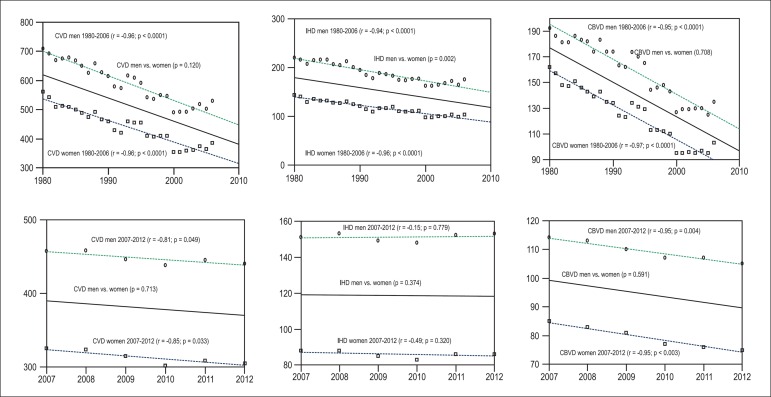
Analysis of the simple linear regression and comparison between the lines of regression of the mortality from cardiovascular diseases (CVD), ischemic heart diseases (IHD), and cerebrovascular diseases (CBVD) between men and women, for the periods of 1980 to 2006 and 2007 to 2012.

The annual alterations in mortality for the periods of 1980-2007 and 2007-2012 were respectively: CVD total: -1.5% e -0.8%, CVD men: -1.4% e -0.6%, CVD women: -1.7% e -1.0%, IHD total: -0.90% e -0.08%, IHD men: -1.1% e 0.1%, IHD women: -1.5% e -0.4%, CBVD total: -1.26% e -1.60%, CBVD men: -1.7% e -1.4% and CBVD women: -2.0% e -1.9%. From 1980 to 2006, there was a decrease in mortality from CVD, IHD, and CBVD in men and women (p < 0.001 for all comparisons) (Figure 2). Results of the linear regression for the period of 1980-2006 were: CVD total: y = 625.9-7.8 (R^2^ = 0.40. p < 0.001), CVD men: y = 707.5-8.4 (R^2^ = 0.91. p < 0.001), CVD women: y = 544.2-7.3 (R^2^ = 0.92. p < 0.001), IHC total: y = 180.1-2.0 (R^2^ = 0.16. p = 0.003), IHC men: y = 220.8-2.3 (R^2^ = 0.89. p < 0.001), IHC women: y = 140.5-1.7 (R^2^ = 0.91. p < 0.0001), CBVD total: y = 180.1-2.7 (R^2^ = 0.56. p < 0.001), CBVD men: y = 198.1-2.7 (R^2^ = 0.94. p < 0.001), and CBVD women: y = 162.2-2.6 (R^2^ = 0.91. p < 0.001). The comparison of the linear regression lines between men and women showed greater reduction in IHD (p = 0.002) in men, but no significant reduction for CVD (p = 0.120) and CBVD (p = 0.708) (Figure 2).

From 2007 to 2012, there was reduction in mortality from CVD and CBVD in men and women, and the results of the linear regressions for this period were: CVD total: y = 394.2-4.1 (R^2^ = 0.01. p =0.753), CVD men: y = 460.1-3.7 (R^2^ = 0.66. p = 0.049), CVD women: y = 329-4.5 (R^2^ = 0.72. p = 0.033), CBVD total: y = 101.3-2.1 (R^2^ = 0.05. p =0.495), CBVD men: y = 116.4-1.9 (R^2^ = 0.90. p = 0.004), and CBVD women: y = 86.7-2.1 (R^2^ = 0.90. p = 0.003). However, no significant alterations in IHD mortality were observed: IHD total: y = 119.3-.25 (R^2^ = 0.02. p = 0.978), IHD men: y = 151 + 0.04 (R^2^ = 0.15. p = 0.779), IHD women: y = 87.7-.54 (R^2^ = 0.24. p = 0.320). The comparisons of linear regressions between men and women did not show differences in mortality trends for CVD (p = 0.713), IHD (p = 0.374) e CBVD (p = 0.591).

## Discussion

This study showed a trend in the reduction of mortality from cardiovascular diseases from 1980 to 2012, but in the analysis of the period of 2007-2012 no reduction in mortality from IHD was observed in men or women.

Studies showed a trend in the reduction of mortality from cardiovascular diseases in several countries, especially in the more developed countries of Western Europe, USA, and Canada.^8-10^ A recent update showed significant reduction in mortality from CVDs in all states of the USA.^10^ However, only a small reduction in mortality from IHD was observed in young adults, especially women.^11^ Despite the significant discrepancies between death rates from CVD in Europe, many European countries also had an increase or small reduction in mortality from CVD.^12^ Control of the risk factor, and improvements in clinical and interventional treatments are the main justifications for the reduction in mortality in more developed countries.^13,14^ Just as in our study, a reduction of mortality from such diseases was also observed in developing countries.^15^ Even with population changes in these countries, such as increase and aging of the population, the trends in mortality from CVD have been maintained with the adjustment of the coefficient for age, gender and specific disease (IHD or CBVD).^9^ Our study showed significant and constant reduction in the mortality from CBVD in the period of 1980 to 2012. This was, most likely, due to an increased facility in diagnosis and treatment of the main risk factor for such diseases - the systemic arterial hypertension (SAH). In 2013, the diagnosis of SAH was 21.2% for the population over 18 years old and >50% for individuals over 65 years old. Almost 70% of these patients with SAH had some kind of medical assistance and 36% took at least one medication for hypertension in the Brazilian government program *Programa Farmácia Popular* (Popular Pharmacy Program).^16^

On the other hand, the complexity of the factors involved in the pathophysiology of the atherosclerosis process largely increases the challenge of preventing IHDs. SAH control has great impact on the morbidity and mortality of CBVDs, while diagnosis and treatment of IHDs involve other risk factors, such as dyslipidemia, smoking, and diabetes, many times unknown until the first coronary event. Associated to the complexity of clinical treatment, there is the limited availability of interventional treatment, restricted to large urban centers. The result is the big heterogeneity of the risk of death from acute myocardial infarction in the different regions of Brazil.^17^ However, in the period between 2007 and 2012, the justifications for a halt in the downward trend in mortality from IHD are unknown. Socioeconomic aspects and decreased access to adequate healthcare system by the less privileged population for diagnosis and treatment of IHDs may be impacting on the change observed in the trend of mortality from such diseases. Baena et al.^18^ have shown an increase in mortality from IHD in the North and Northeast regions, which are known to be underprivileged in Brazil. In mortality from CVD,^19-22^ studies have also shown the significance of social inequalities and discrepancies in level of education of the population. Half of the causes of death from CVD, before 65 years of age, can be attributed to poverty. Likewise, low levels of education contribute to poverty, which further increases the rate of mortality from CVD. Malnourishment, little physical activity, alcohol consumption and smoking are other important risk factors for CVD, and are more prevalent in less privileged layers of the population.^23,24^ Therefore, the institution of public policies of prevention of CVD should be intensified to reinstate a downward trend in mortality from CVD. Environmental, occupational, behavioral and metabolic factors were responsible for almost 90% of disability-adjusted life years (DALYs) and deaths from CVD.^25^

The limitations of this study relate to the quality of the Brazilian data about mortality, such as errors related to diagnosis and precision of death certificates, deaths associated with unknown causes and data entry errors. The number of death certificates containing a diagnosis for the cause of death, such as poorly defined symptoms, signs and health conditions is an indirect indicator of the standard quality of data. These certificates, despite progressive improvement, are still significant in the Northeast, north and Midwest regions in Brazil, but not in the South or Southeast.^26-27^ Validation studies for the data about mortality are not available in the majority of states and cities in Brazil.

## Conclusion

As opposed to CBVD, the trend in mortality from IHD stopped going down in Brazil in the last six years. It is necessary to intensify healthcare policies about the control of the main risk factors so as to reinstate the downward trend in mortality rate from IHD.
